# Characterization of Bimetallic Fe-Ru Oxide Nanoparticles Prepared by Liquid-Phase Plasma Method

**DOI:** 10.1186/s11671-016-1557-8

**Published:** 2016-07-26

**Authors:** Sung-Jin Lee, Heon Lee, Ki-Joon Jeon, Hyunwoong Park, Young-Kwon Park, Sang-Chul Jung

**Affiliations:** 1 Department of Environmental Engineering, Sunchon National University, 255 Jungang-ro, Sunchon, Jeonnam, 540-950 Republic of Korea; 2 Department of Environmental Engineering, Inha University, 100 inharo, Nam-gu, Incheon, 402-751 Republic of Korea; 3 School of Energy Engineering, Kyungpook National University, 80 Daehakro, Bukgu, Daegu, 702-701 Korea; 4 School of Environmental Engineering, University of Seoul, 163 Seoulsiripdaero, Dongdaemun-gu, Seoul, 130-743 Republic of Korea

**Keywords:** Liquid-phase plasma, Bimetallic nanoparticle, Fe, Ru, Reduction potential

## Abstract

The bimetallic Fe-Ru oxide nanoparticles were synthesized in the liquid-phase plasma (LPP) method which employed iron chloride and ruthenium chloride as precursors. The active species (OH·, H_α_, H_β_, and O^I^) and the iron and ruthenium ions were observed in the plasma field created by the LPP process. The spherical-shaped bimetallic Fe-Ru oxide nanoparticles were synthesized by the LPP reaction, and the size of the particles was growing along with the progression of the LPP reaction. The synthesized bimetallic Fe-Ru oxide nanoparticles were comprised of Fe_2_O_3_, Fe_3_O_4_, RuO, and RuO_2_. Ruthenium had a higher reduction potential than iron and resulted in higher ruthenium composition in the synthesized bimetallic nanoparticles. The control of the molar ratio of the precursors in the reactant solution was found to be employed as a means to control the composition of the elements in bimetallic nanoparticles.

## Background

Bimetallic catalysts have been studied by many researchers, and as a result of such studies, they have been applied to many industrial catalytic processes [1, 2]. In general, the bimetallic catalysts have higher performance than the monometallic catalysts [3, 4]. Among diverse bimetallic catalysts developed so far, the Fe-Ru catalysts are the representative alloy system successfully used in the Fischer-Tropsch synthesis [5]. The Fe-Pt catalysts have been known since their methanol activity and selectivity would be changed by the varied proportion in the alloy phase [6]. In addition, the increase of Fe content in zeolite-supported Pd catalysts has been known that it could increase the rate of methanol formation [7]. In this way, the bimetallic catalysts of diverse characteristics can be produced by varying the composition of the second metal component.

On the other hand, the ruthenium oxide (RuO_2_) nanoparticles are added to carbonaceous materials as an electrode material to improve the performance of electrochemical capacitor [8–10]. However, owing to the price of the expensive RuO_2_, the cheaper iron oxide nanoparticles are occasionally added thereto instead [11]. Therefore, if the manufacturing of Fe-Ru bimetallic nanoparticles would be enabled, then it would be presumable that they could be applicable as an electrode material of the electrochemical capacitor.

Recently, the liquid-phase plasma (LPP) process which enabled to control the size and morphology of particles has been spotlighted as a way to produce nanoparticles [12, 13]. The LPP process can produce diverse metals and metal oxide nanoparticles simply by the reduction reaction using electrons and ions which are to be generated in an aqueous solution by plasma [14]. In our previous studies, the cases of successful synthesis of diverse metal nanoparticles including iron nanoparticles through the use of LPP process are reported [15–17]. In addition, the result of improved performance of the electrochemical capacitor electrode produced by employing the nanoparticles synthesized through the LPP process is also reported [18].

In this study, the synthesis of bimetallic Fe-Ru oxide nanoparticles using LPP is reported as a basic advanced study for the application of such nanoparticles to bimetallic catalyst and electrochemical capacitor electrode. The influence of LPP process parameters on the size, morphology, and chemical composition of bimetallic nanoparticles was thus also examined. In addition, the chemical and physical properties of the synthesized bimetallic nanoparticles were analyzed by using several kinds of instrumental analysis.

## Methods

### Materials and Experimental Equipment

Iron chloride tetrahydrate (FeCl_2_·4H_2_O; Kanto Chemical Co.) and ruthenium chloride hydrate (RuCl_3_∙XH_2_O, 45 wt% Ru; Sigma-Aldrich) were used in this study as precursors of Fe metal and Ru metal, respectively. To prevent the coagulation of the particles created by the LPP reaction in the aqueous solution, cetyltrimethylammonium bromide (CTAB; CH_3_(CH_2_)15 N(CH_3_)_3_Br; Daejung Chemicals & Metals) was used as a dispersant. Ultrapure water was employed in this study as a solvent for all applications. The LPP system exploited the power supply (Nano Technology Inc.; NTI-500 W) of high-frequency bipolar pulse which was also used in this study to produce particles from the precursors. The employed LPP system was identical to the one employed in our previous studies [15–17] from which the details of the employed LPP system can be referred to. The conditions of the voltage, frequency, and pulse width set for the creation of plasma were fixed as 250 V, 30 kHz, and 5 μ, respectively.

### Preparation of Composite

The aqueous reaction solution employed to produce the Fe-Ru oxide bimetallic nanoparticles from the LPP reaction was prepared by following the ways. The aqueous solution of pH 2 was prepared by adding 0.1 N HCl to ultrapure water, and the ruthenium chloride and iron chloride were added thereto additionally with ratios of 1:9, 1:4, and 2:3, respectively, to attain a concentration 5 mM of the total metal precursor. And further, CTAB with a 40 % molar ratio with respect to the total precursor quantity (2 mM) was added and then agitated to dissolve it completely. The amount of final aqueous reaction solution used for the test conducted in this study was 300 mL. The prepared aqueous reaction solution was then put into the LPP reactor to induce LPP reaction to generate the Fe-Ru oxide nanoparticles in the solution.

### Structural Characterization

Several chemical active species generated in the Fe-Ru chloride reactant solution by the LPP discharge were observed by using the optical emission spectrometer (OES; Avantes). And the pH of aqueous reaction solution varied by the LPP reaction was measured by using a pH meter (HM-30R; TOA-DKK). The morphology, size, crystal structure, and lattice of the particles generated from metal precursors by reduction were observed through the high-resolution field-emission transmission electron microscope (HR-FETEM; JEM-2100F; JEOL Ltd.). The composition of the elements comprised in the bimetallic nanoparticles was analyzed by using the energy-dispersive spectroscopy (EDS) attached to the HR-FETEM. And to look into the chemical state of iron and ruthenium which comprised the particles, the high-resolution X-ray photoelectron spectroscopy (HR-XPS; Multilab 2000 system; SSK) was used.

## Results and Discussion

### Optical Emission Spectra

**Fig. 1:**
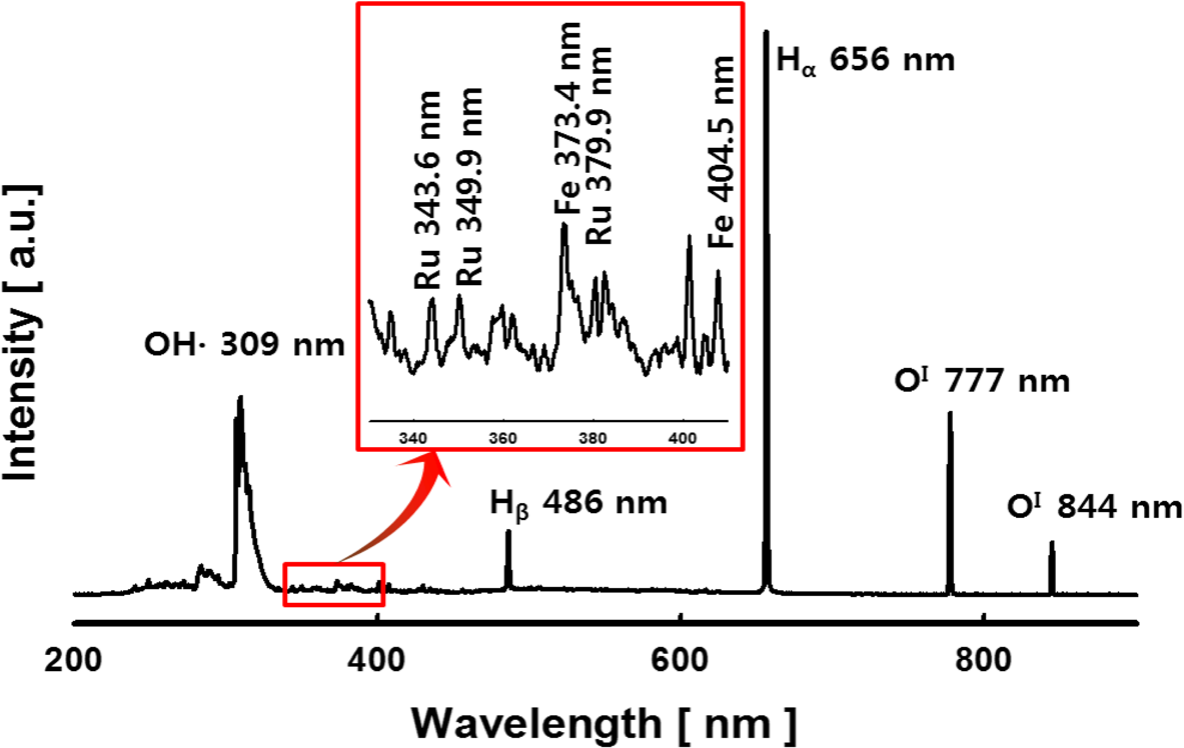
Spatially and temporally integrated emission spectra for the plasma discharge in iron-ruthenium chloride solution

Several chemical active species are created by the discharge of plasma to the reactant solution containing the precursors. And the LPP reaction can be understood by the observation of optical emission spectrum (OES) of such chemical active species. The spectra which were emitted from the reactant solution prepared by using metal precursors which are comprised of the ion chloride and ruthenium chloride were measured, and the results of this measurement are represented in Fig. 1. The peaks of the active species of OH radical (309 nm), H_α_ (656 nm), H_β_ (486 nm), and O^I^ (777, 844 nm) were observed in the plasma field created by the LPP reaction. In particular, the intensity of H_α_ generated at 656 nm shows bigger values compared to the other active species. Besides, the atomic iron (1s^2^2s^2^2p^6^3s^2^3p^6^3d^6^4s^2^^5^D^4^) at 373.4 and 404.5 nm and the atomic ruthenium (1s^2^2s^2^2p^6^3s^2^3p^6^3d^10^4s^2^4p^6^4d^7^5s 5 F^5^) at 343.6, 349.9, and 379.9 nm were observed. Inside the aqueous reaction solution that caused the plasma spark, the electrical field, oxygen bubbling, overpressure shock waves, and ultraviolet radiation are generated. And in a strong electric field produced by an LPP process, a lot of electrons and numerous chemically active species are created [14]. The metal ions remaining in the ionized state in the reaction solution are reduced by the electrons created by the LPP reaction and then precipitated into metal particles. And further, such metal particles can also be oxidized by the active oxidizing species (OH^*^, O^*^, ^1^O_2_, HO_2_, O^−^_2_, H_2_O_2_, or O_3_) generated by plasma discharge and thereby creating metal oxide particles.

### Effect of LPP Reaction Duration

**Fig. 2:**
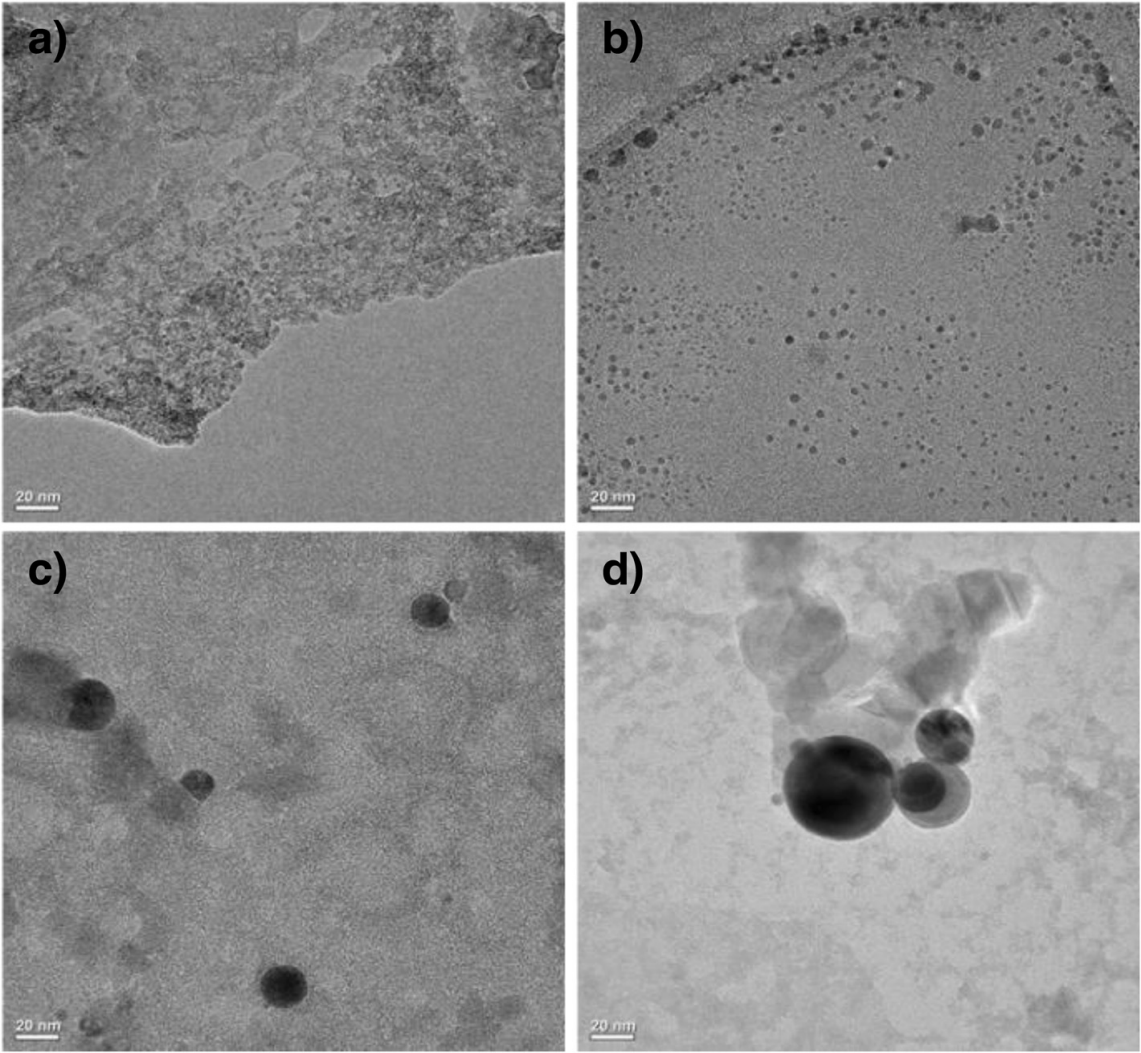
HR-FETEM images of nanoparticles prepared by LPP process with different plasma discharge durations: **a** 10, **b** 20, **c** 40, and **d** 60 min

To observe the morphology of the particles to be created in the aqueous solution by LPP process, the HR-TEM was employed and the results of the observation are represented in Fig. 2. The observed molar ratio between ruthenium chloride and iron chloride was 1:4, and the particles created at the respective reaction durations of 10, 20, 40, and 60 min were observed. Panels a and b represented in Fig. 2 are observations which correspond to each reaction duration of 10 and 20 min, respectively, in which the sizes of the particles of less than 5 nm were observed. And in panel c in Fig. 2, which corresponds to the reaction duration of 40 min, the respective sizes of the particles ranging from 20 to 50 nm were observed. In addition, panel d in Fig. 2 which was the observation of particles created through the duration of 60 min of the LPP process shows particles of more grown size ranged from 50 to 80 nm. Besides, some of the coagulation of particles which arose from the agglomeration of particles which were generated by the reaction duration of 60 min were observed.

### Chemical and Physical Properties of Nanoparticles

**Fig. 3:**
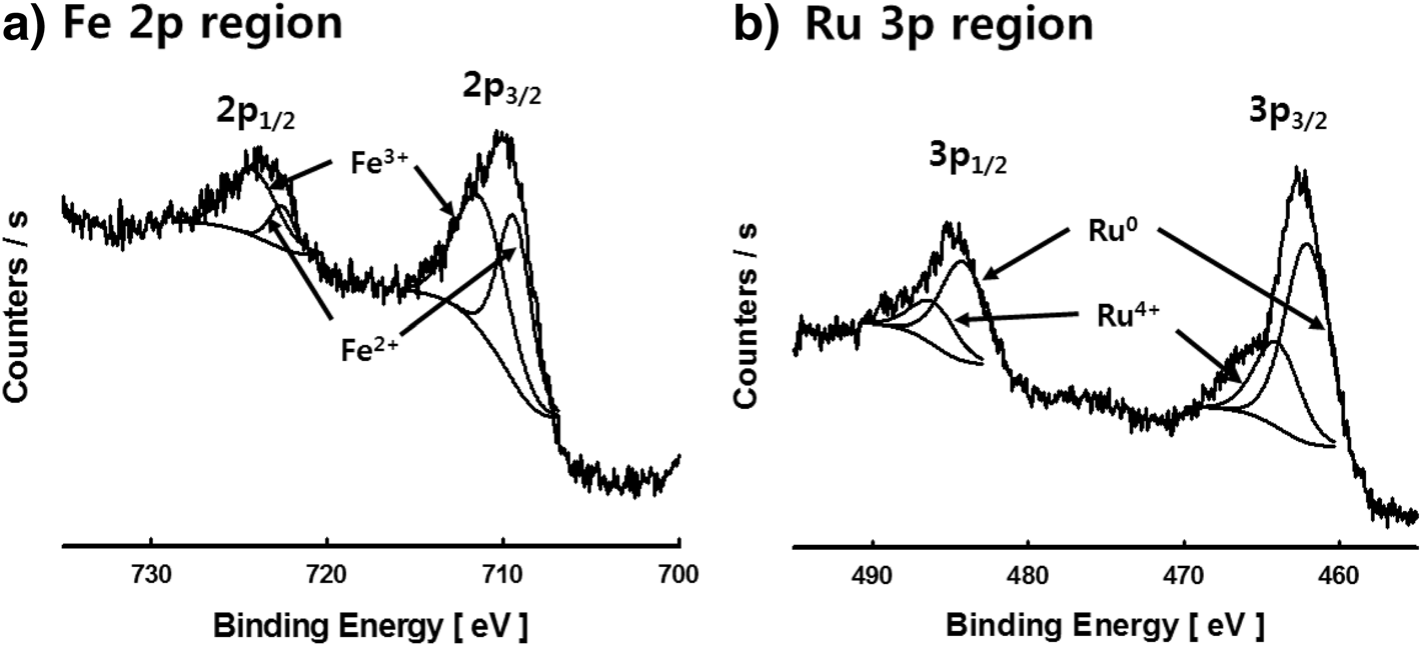
HR-XPS spectra of bimetallic Fe-Ru oxide nanoparticles prepared by the LPP process (**a**, **b**)

To analyze the chemical state of the particles to be created in the RuCl_3_-FeCl_2_ reactant solution by LPP process, the HR-XPS was used and the results attained from the examination are represented in Fig. 3. The particles generated in the aqueous solution were filtered, and the particles collected by the filtering were washed by using the ultrapure water for the observation. The molar ratio between ruthenium chloride and iron chloride contained in the reactant solution was 1:4, and the particles generated by the duration of 60 min of LPP reaction were analyzed. Figure 3a represents the ion element in the 2p region. The 2p_3/2_ iron peak was observed at each point of 709.4 and 711.3 eV while the 2p_1/2_ iron peak was observed from each level of 722.5 and 724.5 eV. The peaks observed from both points of 709.4 and 722.5 eV were of Fe^2+^ while those observed from the points of 710.9 and 724.5 eV were of Fe^3+^. Thus, by the iron peaks observed in the 2p region, the irons synthesized by the LPP reaction were concluded as iron oxides in the forms of Fe_2_O_3_ and Fe_3_O_4_ [19–21]. Figure 3b represents the ruthenium element in the 3p region. The peaks of 3p_3/2_ ruthenium were observed from each level of 462.0 and 463.9 eV, and the peaks of 3p_1/2_ ruthenium were identified from each point of 484.2 and 486.1 eV. The doublet separation of the Ru 3p_3/2_ peak and Ru 3p_1/2_ peak was 22.2 eV, and by which the origination of Ru_2_ and Ru^4+^ was identified. Thus, based on these results, the contemporaneous synthesis of metallic Ru (RuO) and anhydrous RuO_2_ by the LPP reaction was identified [22–25]. From the results obtained by the XPS analysis, the particles synthesized by the LPP process employed in this study were identified as the bimetallic Fe-Ru oxide nanoparticles which were comprised of iron oxide and ruthenium oxide.

**Fig. 4:**
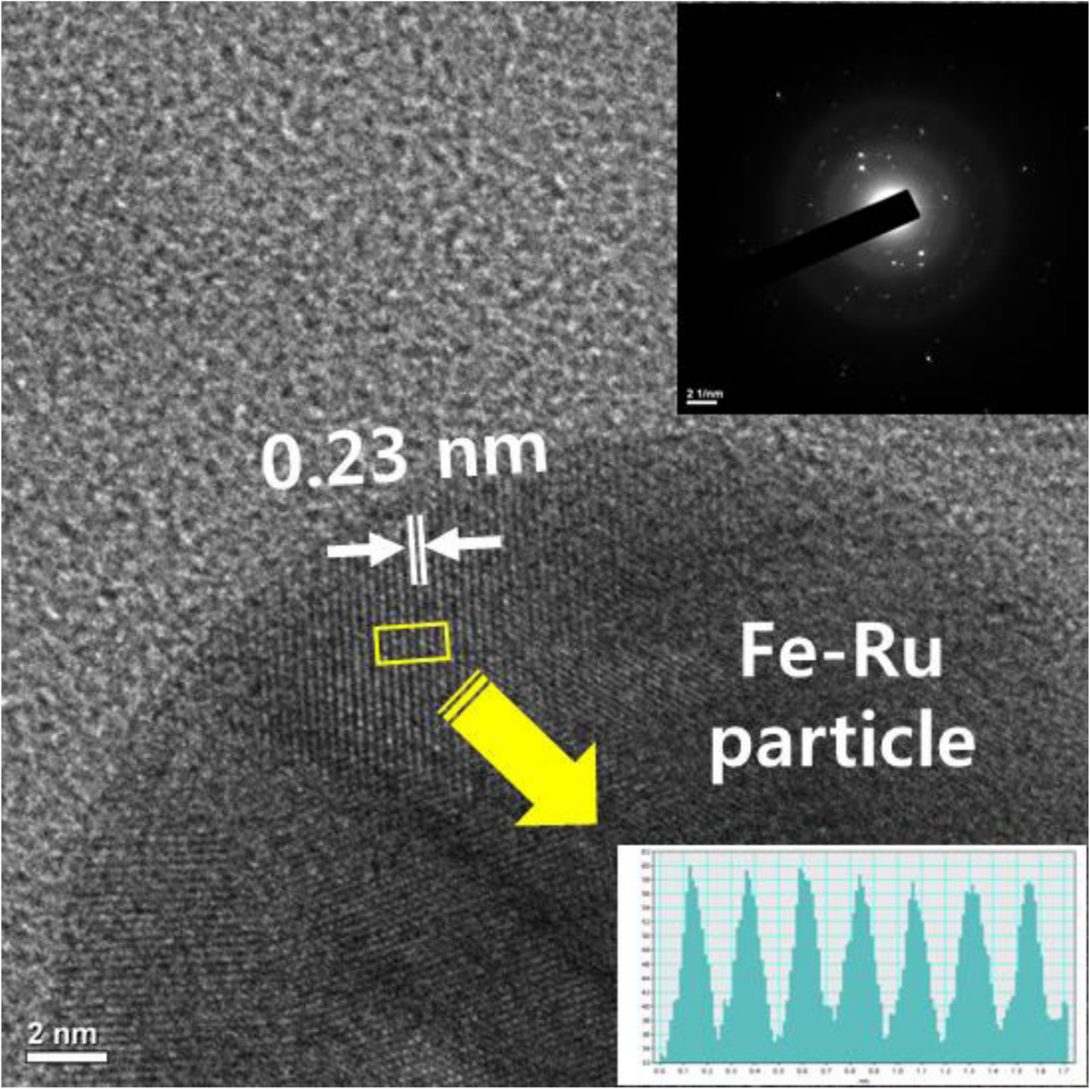
HR-FETEM image and corresponding ED pattern of bimetallic Fe-Ru oxide nanoparticle prepared by the LPP process

To examine the crystal structure of the bimetallic Fe-Ru oxide nanoparticles generated from the RuCl_3_-FeCl_2_ precursor by LPP reaction and remaining in the aqueous solution, they were observed through high magnification of HR-FETEM and the results of the observation are represented in Fig. 4. The molar ratio between ruthenium chloride and iron chloride precursors contained in the reactant solution was 1:4, and the bimetallic Fe-Ru oxide nanoparticles generated by the duration of 60 min of LPP reaction were observed. The lattice fringes on the surface of bimetallic Fe-Ru oxide nanoparticles were identified, and the measurement of crystal lattice spacing was approximately 0.23 nm. Besides, in the upper right region of Fig. 4, the electron discharge (ED) pattern of the bimetallic Fe-Ru oxide nanoparticles is represented. The ED pattern did not show many spots and circles, indicating that the bimetallic Fe-Ru oxide nanoparticles precipitated are very fine amorphous particles.

### Effect of pH

**Fig. 5:**
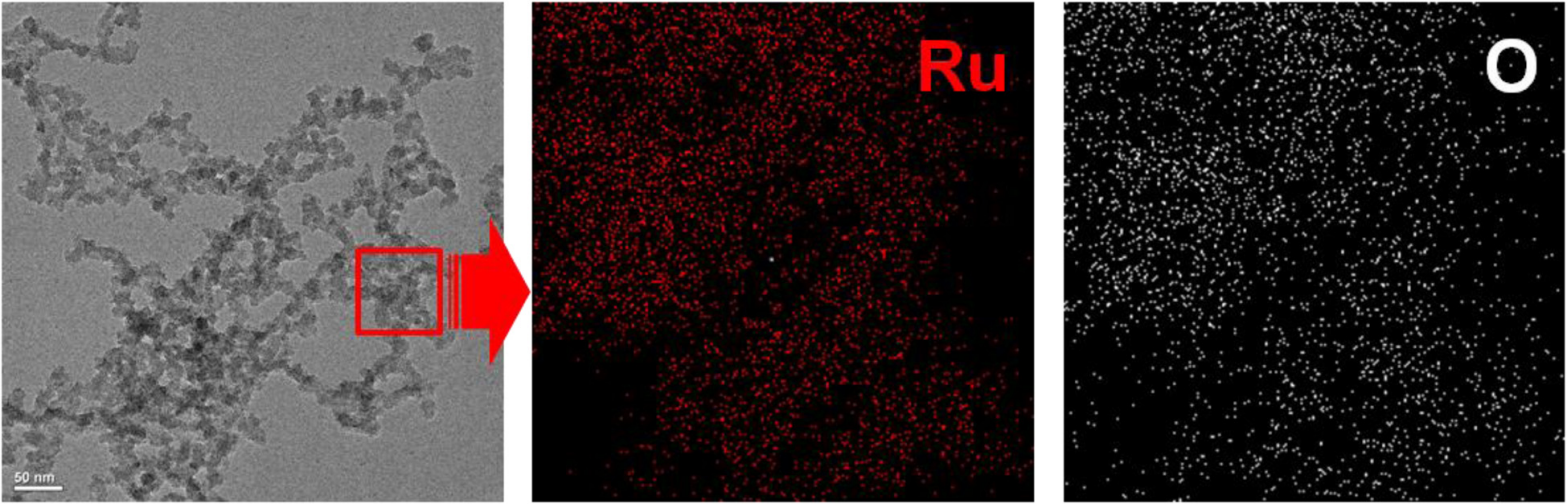
HR-FETEM images of ruthenium oxide nanoparticles precipitated from pH 7 of the reactant solution

The ruthenium chloride employed as a precursor in this study has been known that it would constitute a colloid in the form of ruthenium oxide in a reactant aqueous solution by the changes in pH and temperature [26, 27]. And in this study, the precipitation in the reactant aqueous solution of pH 7 which was prepared through the ultrapure water taken instead of the use of HCl was observed. In Fig. 5, the deposits which were precipitated in the reactant aqueous solution and were observed through HR-FETEM are represented. The precipitates were identified as ruthenium oxides by the results of the used EDS analysis and are represented as an element mapping on the right side of Fig. 5. On the other hand, the formation of precipitate was decreasing along with the lowering of the level of pH in the reactant aqueous solution, and in the reactant aqueous solution of pH 2, the precipitates were not observed.

**Fig. 6:**
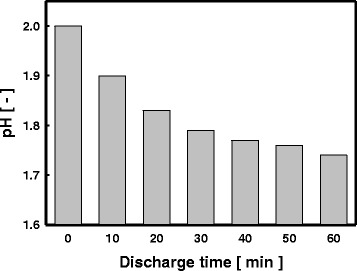
Change in pH of the reactant solution as a function of the LPP discharge time

In this study, to prevent the creation of the colloid of ruthenium precursor, the reactant aqueous solution of pH 2 was prepared by HCl. In Fig. 6, the changes of the pH of the reactant aqueous solution in the duration of LPP reaction are represented. It shows the level of pH tended to be decreasing continuously along with the progression of LPP reaction. In the LPP reaction, as illustrated in Fig. 1, reactive active species (OH radical, H_α_, H_β_, and O^I^) are generated, and in particular, the excited hydrogen would turn into H^+^ by the reaction with metal ions residing in the reactant aqueous solution as expressed in Eq. 1 [28]. Thus, it was estimated that, along with the progression of the LPP reaction, the concentration of H^+^ in the reactant aqueous solution increases and thereby the level of pH of the reactant aqueous solution decreases.

1 $$\mathrm{Meta}{\mathrm{l}}^{+} + \mathrm{H}\bullet \to \mathrm{Meta}{\mathrm{l}}^0+{\mathrm{H}}^{+}$$

### Effect of Precursor Molar Ratio

**Fig. 7:**
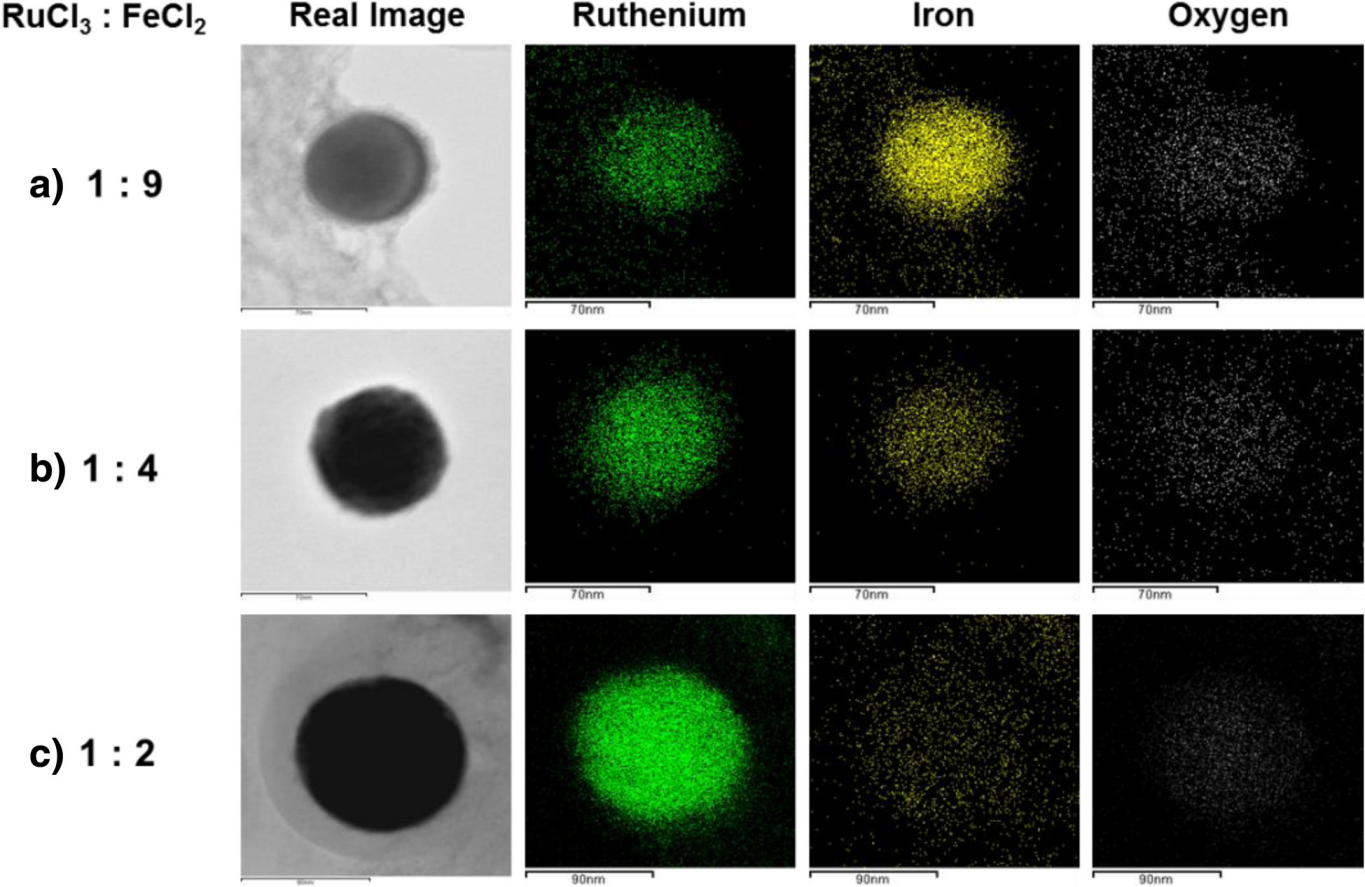
HR-FETEM image and individual element mapping result of bimetallic Fe-Ru oxide nanoparticle prepared by LPP process with different molar ratio of precursor (**a**–**c**)

To examine the composition of the elements comprising the bimetallic Fe-Ru oxide nanoparticles synthesized by the LPP reaction introduced in this study, the EDS analysis was carried out and the results attained from the analysis are presented in Fig. 7. The bimetallic Fe-Ru oxide nanoparticles examined by EDS are those generated from the 60-min duration of the LPP reaction. In the EDS analysis, the molar ratio between ruthenium chloride and iron chloride was varied with respective values of 1:9, 1:4, and 1:2 to examine the influence of the change of molar ratio between precursors on the composition of bimetallic Fe-Ru oxide nanoparticles. The level of the concentration of the total precursors in the reactant solution was kept constantly at 5 mM. In Fig. 5, the ruthenium (green dots), iron (yellow dots), and oxygen (white dots) are displayed together with the real images of bimetallic Fe-Ru oxide nanoparticles observed through the HR-FETEM. Since the ruthenium, iron, and oxygen elements were all detected from one particle, the particles synthesized by the LPP reaction employed in this study were identified as the bimetallic Fe-Ru oxide particles.

In case (a, 1:9) the content of iron precursor is richer than that of ruthenium precursor in the reactant solution, the synthesis of bimetallic Fe-Ru oxide particles of which the level of iron composition is higher than that of ruthenium can be identified from the yellow dots representing an iron. In the case of the molar ratio between ruthenium precursor and iron precursor was 1:2 (c), the synthesis of bimetallic Fe-Ru oxide particles of which the level of ruthenium composition is higher than that of iron can be identified from the relative abundance as the green dots representing ruthenium. Besides, the white dots representing the oxygen element in the synthesized particles were observed from all the cases of the respective conditions; however, the amount of white dots tended to decrease in accordance with the increase of the constituent of ruthenium.

The composition of the elements contained in the bimetallic Fe-Ru oxide nanoparticles were analyzed through the EDS as it was used in Fig. 7, and the results obtained are therefore summarized and represented in Table 1. Along with the increase of the molar ratio of iron chloride to that of ruthenium chloride, the level of iron composition in the bimetallic nanoparticles tended to increase. However, on the other hand, the level of iron composition in the generated bimetallic nanoparticles remained one third that of ruthenium (43.65:14.63, at.%) in the case of the condition of molar ratio of 1:2 between RuCl_3_ and FeCl_2_ despite the doubled amount of iron precursor in the reactant solution. The reduction reactivity of a metal is determined by its ionization tendency and normal potential. Among the metals, the standard potentials of ruthenium and iron are as follows:

2 $$\mathrm{R}{\mathrm{u}}^{3+}\left(\mathrm{a}\mathrm{q}.\right) + 3{\mathrm{e}}^{-}\leftrightarrow\ \mathrm{R}\mathrm{u}\ \left(\mathrm{s}\right)\kern0.75em {\mathrm{E}}^0\left(\mathrm{volts}\right) = +0.60\ \mathrm{V}$$

3 $$\mathrm{F}{\mathrm{e}}^{2+}\left(\mathrm{a}\mathrm{q}.\right) + 2{\mathrm{e}}^{-}\leftrightarrow\ \mathrm{F}\mathrm{e}\ \left(\mathrm{s}\right)\kern0.75em {\mathrm{E}}^0\left(\mathrm{volts}\right) = -0.44\ \mathrm{V}$$

**Table 1:** Chemical composition of the bimetallic Fe-Ru oxide nanoparticles as a different molar ratio of precursor

RuCl_3_:FeCl_2_	Ru		Fe		O	
Molar ratio	wt.%	at.%	wt.%	at.%	wt.%	at.%
1:9	29.47	12.22	51.92	38.98	18.61	48.80
1:4	47.63	22.22	36.43	30.76	15.94	47.01
1:2	69.16	43.65	20.38	14.63	10.46	41.72

Thus, the higher reduction potential and lower ionization tendency of ruthenium than those of iron were identified. Owing to these properties, the ruthenium ions generated by the LPP reaction were reduced ahead of others and thereby rendered higher composition of ruthenium in the synthesized bimetallic nanoparticles. In the meantime, all the bimetallic nanoparticles synthesized under every condition showed that they were containing oxygen elements. This was estimated that it could be attributable to the oxidation of metal particles by active oxidizing species (^1^O_2_, O^−^_2_, O•, OH•, HO_2_, H_2_O_2_, and O_3_) which were generated by the LPP reaction. In our previous study [15], the prevention of an oxidation of iron metal particles into iron oxide particles by ethanol employed as a solvent instead of water for the preparation of reactant solution was identified. And accordingly, the possible preparation of bimetallic Fe-Ru nanoparticles by an application of ethanol as a solvent can also be presumable. However, in this study, the application of ethanol as a solvent was not employed for the synthesis of bimetallic Fe-Ru oxide nanoparticles by taking the usage of Fe-Ru oxide as an electrode material of electrochemical capacitor into account. The preparation of bimetallic Fe-Ru nanoparticles through the use of LPP process will be considered in our future study. In addition, through results obtained from the EDX analysis, the adjustment of molar ratio between precursors contained in the reactant solution was identified that it could change the composition of the elements comprising bimetallic nanoparticles accordingly.

## Conclusions

In this study, the bimetallic Fe-Ru oxide nanoparticles were synthesized through the LPP process. The iron chloride and ruthenium chloride were employed as precursors to produce the reactant aqueous solution of which pH became lower along with the progression of LPP reaction. And in the plasma field created by the LPP reaction, the active species (OH radical, H_α_, H_β_ and O^I^), iron ions, and ruthenium ions were observed. The spherical-shaped bimetallic Fe-Ru oxide nanoparticles of the size ranged from 5 to 80 nm were synthesized by the LPP reaction where the size was growing along with the progression of LPP reaction. The synthesized bimetallic Fe-Ru oxide nanoparticles are thereby comprised iron oxides (Fe_2_O_3_ and Fe_3_O_4_), metallic Ru (Ru^0^), and anhydrous ruthenium oxide (RuO_2_). The molar ratio between precursors contained in reactant solution would be influential on chemical composition of the constituents in bimetallic Fe-Ru oxide nanoparticles; however, the ruthenium ions were reduced ahead of iron ions owing to its comparatively higher reduction potential. And the adjustment of the molar ratio among precursors contained in the reactant solution was found that it could control the composition of the elements which are comprised in the bimetallic nanoparticles.
